# Crystal structure and Hirshfeld surface analysis of 1,3,3,4,4,5,5-hepta­fluoro-2-(3-[(2,3,3,4,4,5,5-hepta­fluoro­cyclo­penten-1-yl)­oxy]-2-{[(2,3,3,4,4,5,5-hepta­fluoro­cyclo­penten-1-yl)­oxy]meth­yl}-2-methyl­prop­oxy)cyclo­pentene

**DOI:** 10.1107/S2056989024011484

**Published:** 2025-01-01

**Authors:** Andrew J. Peloquin, Gary J. Balaich, Abby R. Jennings

**Affiliations:** a Department of Chemistry United States Air Force Academy,Colorado Springs CO 80840 USA; University of Aberdeen, United Kingdom

**Keywords:** crystal structure, perfluoro­cyclo­pentene, fluorine–fluorine inter­action

## Abstract

In the title compound, C_20_H_9_F_21_O_3_, a central *sp*^3^-hybridized carbon atom is decorated with three hepta­fluoro-2-meth­yloxy(cyclo­pent-1-ene) arms and a methyl group. The primary packing is determined by C—F⋯F—C inter­actions, forming [001] chains, which are consolidated *via* weaker C—F⋯F—C and C—H⋯F—C contacts. A Hirshfeld surface analysis was conducted to aid in the visualization of these various influences on the packing: this revealed that the largest contribution to the surface contacts arises from F⋯F inter­actions (53.5%), followed by F⋯H/H⋯F (34.5%) and F⋯C/C⋯F (7.1%).

## Abstract 2

In the title compound, a central *sp*^3^-hybridized carbon atom is decorated with three hepta­fluoro-2-meth­yloxy(cyclo­pent-1-ene) arms and a methyl group. The primary packing is determined by C—F⋯F—C inter­actions, forming [001] chains, which are consolidated *via* weaker C—F⋯F—C and C—H⋯F—C contacts.

## 1. Chemical context

Perfluoro­cyclo­pentene (C_5_F_8_; PFCP) is known to be selectively reactive towards nucleophilic addition at the fluoro­olefin, *via* simultaneous addition at the 1- and 5-positions or controlled to only add at the 1-position (Alvino *et al.*, 2020 ▸). In the former case, the fluorine atom in the 5-position is latently reactive and can be utilized to create more complex fluorinated mol­ecules and materials (Lauer *et al.*, 2024 ▸). As such, the synthesis and single-crystal structure of the title compound, C_20_H_9_F_2_O_3_, is reported herein. This mol­ecule was designed to be tri-functional, in that it contains three latently reactive fluorine atoms, which could be employed in designing more complex materials, such as a dendrimer core or for cross-linking (Abbasi *et al.*, 2014 ▸; Weerasinghe *et al.*, 2023 ▸).
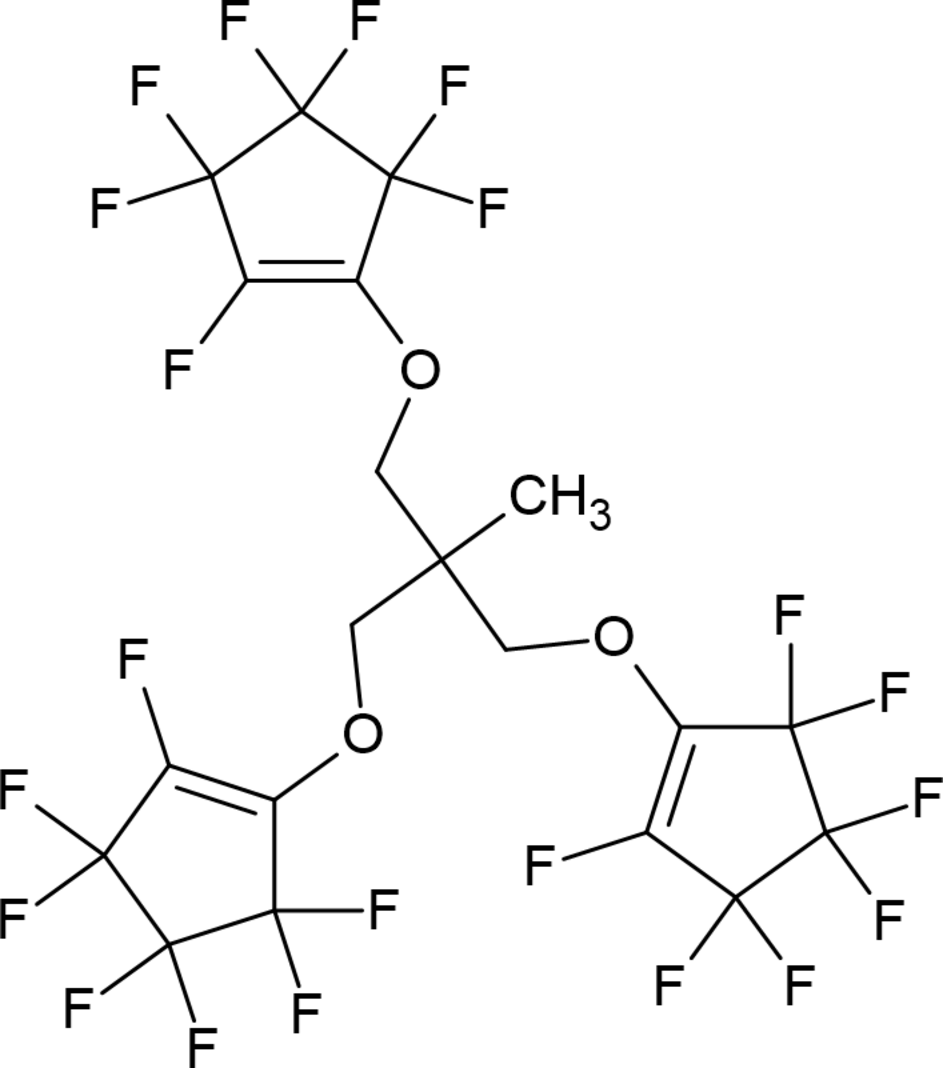


## 2. Structural commentary

**Figure 1:**
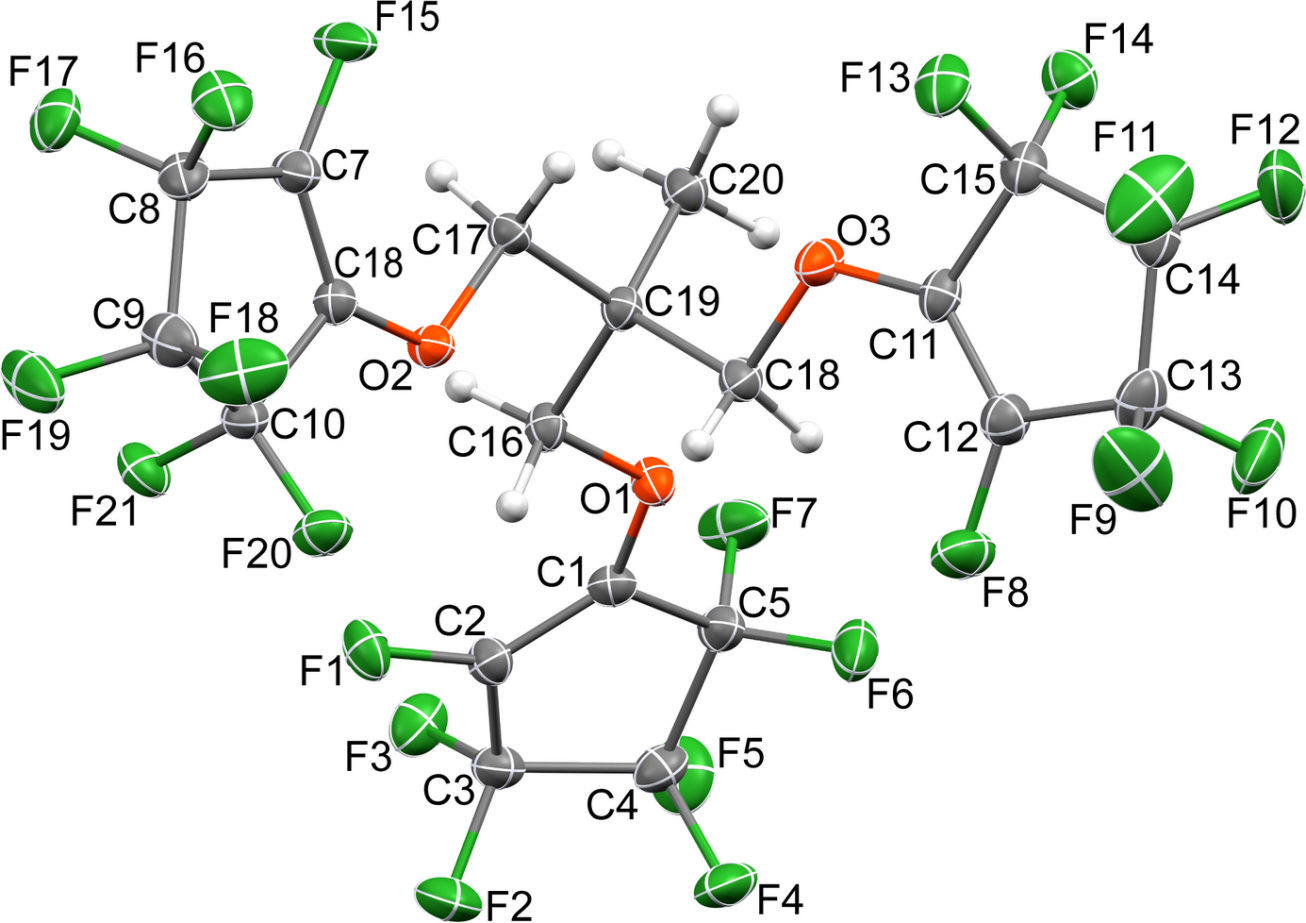
The mol­ecular structure of the title compound. Displacement ellipsoids are shown at the 50% probability level.

The structure of the title highly fluorinated alkyl ether mol­ecule, C_20_H_9_F_21_O_3_, consists of a central *s*p^3^-hybridized carbon atom (C19) covalently bound to a methyl group (C20) and three hepta­fluoro-2-meth­yloxy(cyclo­pent-1-ene) arms (Fig. 1 ▸). The geometry around C19 is nearly that of an ideal tetra­hedral *sp*^3^ geometry, with an average C—C—C bond angle of 109.47^o^. Using a plane defined by C16, C17 and C18, the three non-methyl carbon atoms bound to the *sp*^3^ carbon atom as reference, two of the ether oxygen atoms (O1 and O3) are oriented to the methyl side of the plane, with the third ether oxygen atom (O2) below the plane. Within the cyclo­pentenyl rings, the C=C double-bond lengths range from 1.328 (3) to 1.334 (3) Å. The C—C single bonds to either side of the C=C double bond are approximately 0.15 Å longer, ranging from 1.478 (3) to 1.497 (2) Å. The final two C—C single bonds in the ring are longer yet, ranging from 1.537 (3) to 1.554 (3) Å. The r.m.s. cyclo­pentenyl ring plane angle with respect to the plane defined by its corresponding C_ether_—C*sp*^3^—C_meth­yl_ fragment range from 14.62 (15) to 40.25 (13)°.

## 3. Supra­molecular features

**Figure 2:**
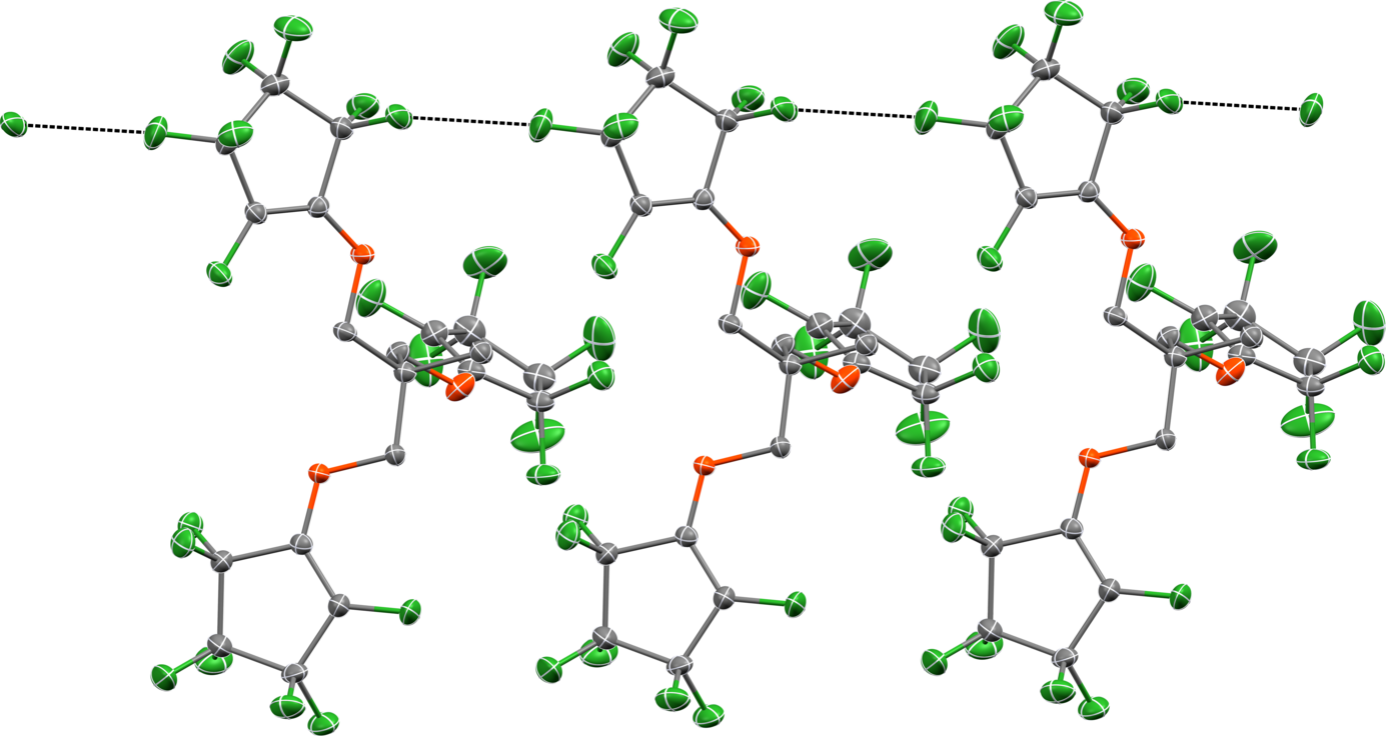
Part of the packing of the title compound showing chains formed by short C—F⋯F—C inter­actions, propagating along the *a-*axis direction.

The primary directional inter­action in the extended structure occurs as a short C—F⋯F—C inter­action between F2 and F7^i^ [symmetry code: (i) *x* − 1, *y*, *z*] at 2.5983 (16) Å, compared to a van der Waals separation of about 2.94 Å. This inter­action contributes to the formation of chains propagating along the *a-*axis direction (Fig. 2 ▸). The packing is consolidated in the crystallographic *c*-axis direction *via* a weak C—F⋯F—C inter­action between F7 and F13^ii^ [symmetry code: (ii) *x*, 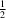 − *y*, 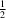 + *z*] at 2.6848 (15) Å and in the *b*-axis direction by a C—H⋯F hydrogen bond between C17—H17*A* and F9^iii^ [symmetry code: (iii) 1 − *x*, −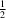 + *y*, 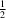 − *z*] at 2.546 (2) Å [angle = 127.61 (14)°].

**Figure 3:**
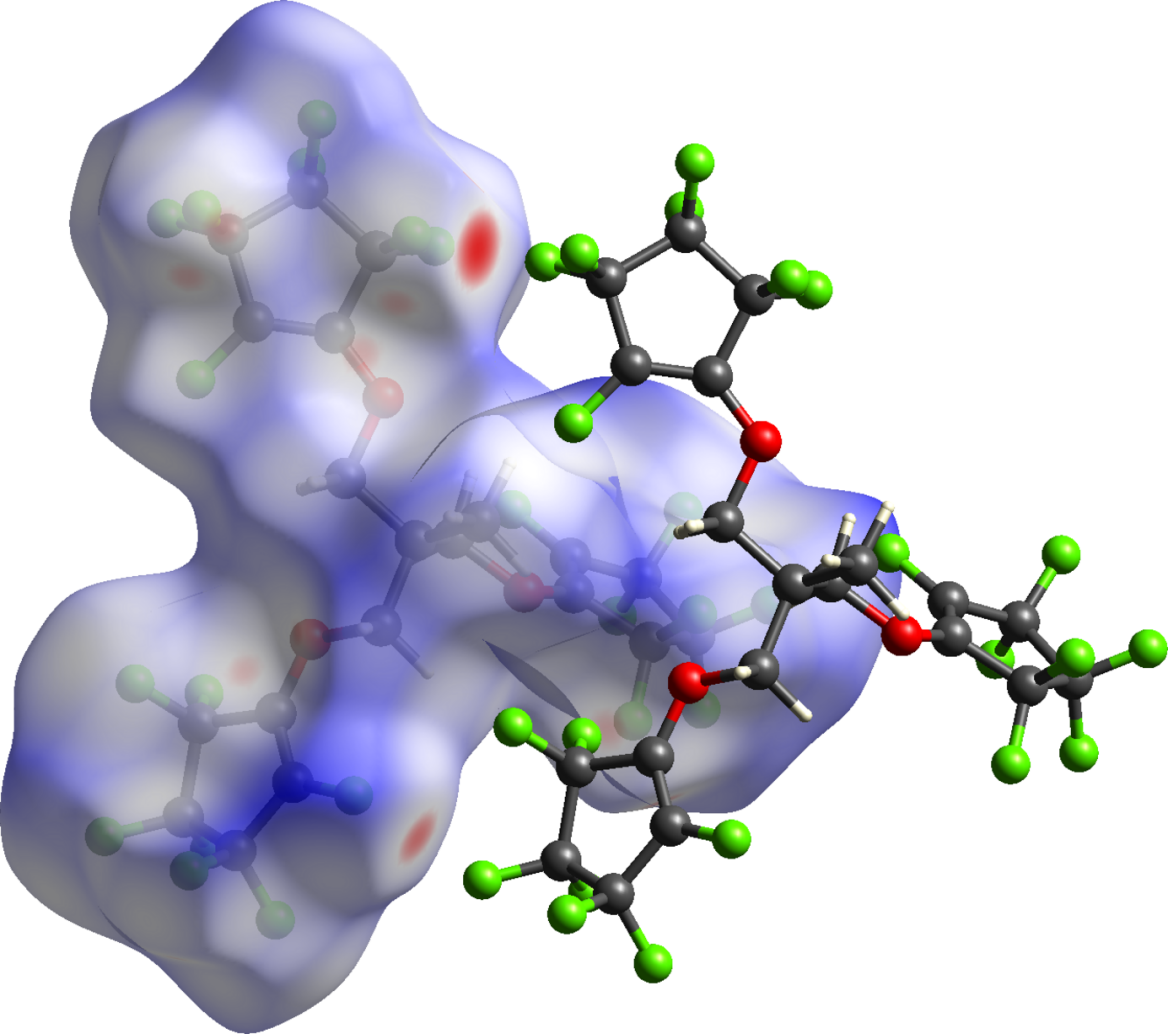
Map of *d*_norm_ onto the Hirshfeld surface for the title compound.

Hirshfeld surface analysis was used to investigate the presence of hydrogen bonds and inter­molecular inter­actions in the crystal structure. The Hirshfeld surface analysis (Spackman & Jayatilaka, 2009 ▸) and the associated two-dimensional fingerprint plots (Spackman & McKinnon, 2002 ▸) were generated by *CrystalExplorer17.5* (Turner *et al.*, 2017 ▸), using a standard surface resolution. The pale-red spots symbolize short contacts and negative *d*_norm_ values on the corresponding surface plots shown in Fig. 3 ▸, associated with their relative contributions to the Hirshfeld surface.

**Figure 4:**
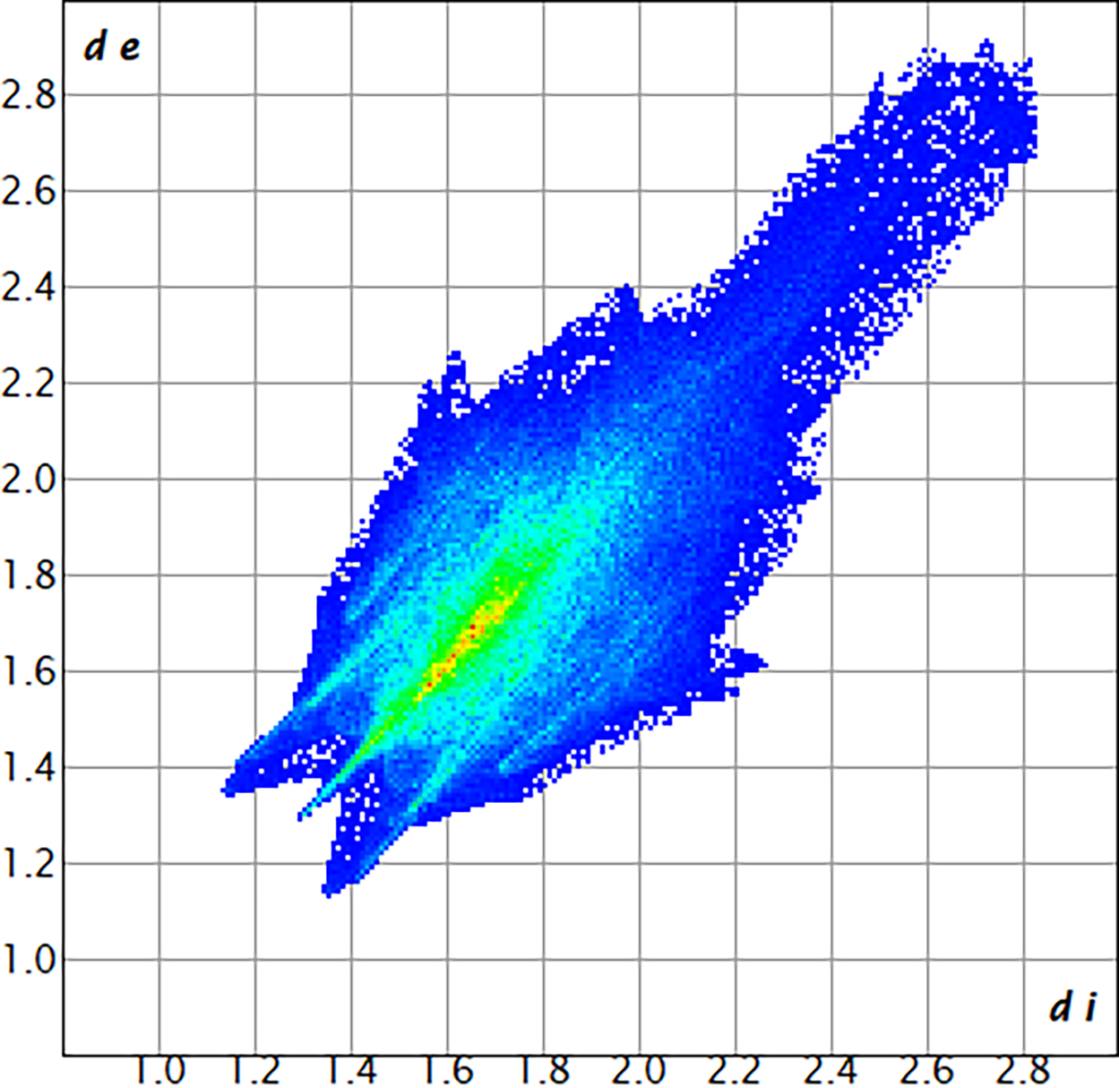
The overall two-dimensional fingerprint plot for the title compound.

The largest contribution to the overall crystal packing in the title compound is from F⋯F inter­actions (53.3%), represented as a single, central spike on the fingerprint plot at 1.30 Å < (*d*_i_ + *d*_e_) < 1.35 Å (Fig. 4 ▸, Table 1 ▸). A significant portion of the inter­molecular inter­actions can also be attributed to F⋯H/H⋯F inter­actions (34.5%), visible on the fingerprint plot as a pair of spikes at 1.15 Å < (*d*_i_ + *d*_e_) < 1.35 Å. The remaining 12% of the inter­actions are attributed to F⋯C/C⋯F, F⋯O/O⋯F, and H⋯H contacts.

## 4. Database survey

A search of the November 2023 release of the Cambridge Structure Database (CSD; Groom *et al.*, 2016 ▸), with updates through September 2024, was performed using the program *ConQuest* (Bruno *et al.*, 2002 ▸). A for search the perfluoro­cyclo­pentene-ether moiety yielded one result, 4,4′-bis­[(2,3,3,4,4,5,5-hepta­fluoro­cyclo­pent-1-en-1-yl)­oxy]biphenyl (CSD refcode GILXUW; Sharma *et al.*, 2013 ▸). This compound contains a pair of perfluoro­cyclo­pentenyl rings bound through an ether linkage to a 4,4′-bis­phenol aromatic core. A short ring C=C bond is observed, flanked by two medium length C—C bonds and two long C—C bonds to complete the ring, similar to the pattern observed in the title compound. A search for the non-fluorinated analog yielded four results. While the C=C double bond is clearly observed, the remaining four C—C bonds within the ring are more similar in bond length to one another.

## 5. Synthesis and crystallization

The title compound was prepared by a modified literature procedure (Alvino *et al.*, 2020 ▸) using di­methyl­formamide (DMF) (20 ml), perfluoro­cyclo­pentene (3.0 ml, 22.4 mmol), tri­methyl­olethane (0.81 g, 6.7 mmol), and tri­ethyl­amine (2.8 ml, 20.2 mmol). The isolated compound (1.0 g) was purified using a plug of silica gel and 80 ml of a 1:3 hexa­ne:ethyl acetate solution. All volatiles were removed under reduced pressure and the compound was obtained as a faint yellow, waxy solid (0.57 g, 57%). Crystals of the title compound, in the form of faint-yellow rectangular prisms, were obtained by slow evaporation from diethyl ether solution. ^1^H NMR (500 MHz, CDCl_3_): δ 4.37 (*d*, –C*H_2_*–, 6H, *J*_HF_ = 2.5 Hz), 1.25 (*s*, –C*H_3_*, 3H); ^19^F NMR (470 MHz, CDCl_3_): δ −115.2 (*d*, 6F, *J*_FF_ = 12.7 MHz), −115.8 (*d*, 6F, *J*_FF_ = 10.8), −129.4 (*s*, 6F), −158.2 (*bs*, 3F); ^13^C NMR (125 MHz, CDCl_3_): δ 72.9 {*d*, –[(–*C*H_2_)_3_CCH_3_], *J*_CF_ = 4.5 Hz], 41.3 [–(CH_2_)_3_*C*CH_3_], 15.8 [–(CH_2_)_3_C*C*H_3_].

## 6. Refinement

Crystal data, data collection and structure refinement details are summarized in Table 2 ▸. The coordinates of all H atoms were freely refined.

## Supplementary Material

Crystal structure: contains datablock(s) I. DOI: 10.1107/S2056989024011484/hb8112sup1.cif

Structure factors: contains datablock(s) I. DOI: 10.1107/S2056989024011484/hb8112Isup2.hkl

Supporting information file. DOI: 10.1107/S2056989024011484/hb8112Isup3.cml

CCDC reference: 2405292

Additional supporting information:  crystallographic information; 3D view; checkCIF report

## Figures and Tables

**Table 1:** Percentage contribution of inter-atomic contacts to the Hirshfeld surface for the title compound

Contact	Percentage contribution
F⋯F	53.5
F⋯H/H⋯F	34.5
F⋯C/C⋯F	7.1
F⋯O/O⋯F	3.0
H⋯H	2.1

**Table 2:** Experimental details

Crystal data	
Chemical formula	C_20_H_9_F_21_O_3_
*M*_r_	696.26
Crystal system, space group	Monoclinic, *P*2_1_/*c*
Temperature (K)	100
*a*, *b*, *c* (Å)	7.0271 (1), 19.2243 (1), 17.7558 (1)
β (°)	97.332 (1)
*V* (Å^3^)	2379.04 (4)
*Z*	4
Radiation type	Cu *K*α
μ (mm^−1^)	2.21
Crystal size (mm)	0.23 × 0.09 × 0.07

Data collection	
Diffractometer	XtaLAB Synergy, Dualflex, HyPix3000
Absorption correction	Gaussian [*CrysAlis PRO*; Rigaku OD, 2019 ▸)
*T*_min_, *T*_max_	0.681, 1.000
No. of measured, independent and observed [*I* ≥ 2u(*I*)] reflections	23552, 4403, 4160
*R*_int_	0.020
(sin θ/λ)_max_ (Å^−1^)	0.605

Refinement	
*R*[*F*^2^ > 2σ(*F*^2^)], *wR*(*F*^2^), *S*	0.037, 0.088, 1.07
No. of reflections	4403
No. of parameters	398
H-atom treatment	H-atom parameters constrained
Δρ_max_, Δρ_min_ (e Å^−3^)	0.90, −0.49

Computer programs: *CrysAlis PRO* (Rigaku OD, 2019 ▸), *SHELXT2018/2* (Sheldrick, 2015*a* ▸), *SHELXL2018/3* (Sheldrick, 2015*b* ▸), *Mercury* (Macrae *et al.*, 2008) and *OLEX2* (Dolomanov *et al.*, 2009 ▸).

## supplementary crystallographic information

### 1,3,3,4,4,5,5-Heptafluoro-2-(3-[(2,3,3,4,4,5,5-heptafluorocyclopenten-1-yl)oxy]-2-{[(2,3,3,4,4,5,5-heptafluorocyclopenten-1-yl)oxy]methyl}-2-methylpropoxy)cyclopentene Crystal data

**Table:**

C_20_H_9_F_21_O_3_	*F*(000) = 1376.511
*M**_r_* = 696.26	*D*_x_ = 1.944 Mg m^−^^3^
Monoclinic, *P*2_1_/*c*	Cu *K*α radiation, λ = 1.54184 Å
*a* = 7.0271 (1) Å	Cell parameters from 18314 reflections
*b* = 19.2243 (1) Å	θ = 3.4–68.7°
*c* = 17.7558 (1) Å	µ = 2.21 mm^−^^1^
β = 97.332 (1)°	*T* = 100 K
*V* = 2379.04 (4) Å^3^	Needle, colourless
*Z* = 4	0.23 × 0.09 × 0.07 mm

### 1,3,3,4,4,5,5-Heptafluoro-2-(3-[(2,3,3,4,4,5,5-heptafluorocyclopenten-1-yl)oxy]-2-{[(2,3,3,4,4,5,5-heptafluorocyclopenten-1-yl)oxy]methyl}-2-methylpropoxy)cyclopentene Data collection

**Table:**

XtaLAB Synergy, Dualflex, HyPix3000 diffractometer	4403 independent reflections
Radiation source: micro-focus sealed X-ray tube, PhotonJet (Cu) X-ray Source	4160 reflections with *I*≥ 2u(*I*)
Mirror monochromator	*R*_int_ = 0.020
Detector resolution: 10.0000 pixels mm^-1^	θ_max_ = 68.9°, θ_min_ = 3.4°
φ and ω scans	*h* = −8→8
Absorption correction: gaussian [CrysAlisPro; Rigaku OD, 2019)	*k* = −23→21
*T*_min_ = 0.681, *T*_max_ = 1.000	*l* = −21→21
23552 measured reflections	

### 1,3,3,4,4,5,5-Heptafluoro-2-(3-[(2,3,3,4,4,5,5-heptafluorocyclopenten-1-yl)oxy]-2-{[(2,3,3,4,4,5,5-heptafluorocyclopenten-1-yl)oxy]methyl}-2-methylpropoxy)cyclopentene Refinement

**Table:**

Refinement on *F*^2^	13 constraints
Least-squares matrix: full	Primary atom site location: dual
*R*[*F*^2^ > 2σ(*F*^2^)] = 0.037	H-atom parameters constrained
*wR*(*F*^2^) = 0.088	*w* = 1/[σ^2^(*F*_o_^2^) + (0.0336*P*)^2^ + 2.693*P*] where *P* = (*F*_o_^2^ + 2*F*_c_^2^)/3
*S* = 1.07	(Δ/σ)_max_ = 0.001
4403 reflections	Δρ_max_ = 0.90 e Å^−^^3^
398 parameters	Δρ_min_ = −0.49 e Å^−^^3^
0 restraints	

### 1,3,3,4,4,5,5-Heptafluoro-2-(3-[(2,3,3,4,4,5,5-heptafluorocyclopenten-1-yl)oxy]-2-{[(2,3,3,4,4,5,5-heptafluorocyclopenten-1-yl)oxy]methyl}-2-methylpropoxy)cyclopentene Fractional atomic coordinates and isotropic or equivalent isotropic displacement parameters (Å^2^)

**Table:**

	*x*	*y*	*z*	*U*_iso_*/*U*_eq_	
F1	0.16671 (15)	0.06887 (6)	0.41851 (6)	0.0265 (3)	
F2	0.05627 (16)	0.07023 (7)	0.56596 (7)	0.0360 (3)	
F3	0.26356 (18)	−0.01232 (6)	0.56315 (6)	0.0303 (3)	
F4	0.29905 (18)	0.15059 (7)	0.64289 (7)	0.0370 (3)	
F5	0.4531 (2)	0.05472 (7)	0.67162 (7)	0.0394 (3)	
F6	0.60320 (18)	0.18574 (7)	0.58931 (7)	0.0332 (3)	
F7	0.69217 (16)	0.07832 (7)	0.57776 (7)	0.0364 (3)	
F8	0.54974 (18)	0.40300 (6)	0.39070 (8)	0.0365 (3)	
F9	0.6667 (2)	0.53068 (7)	0.32545 (11)	0.0556 (4)	
F10	0.8592 (2)	0.50227 (8)	0.42497 (8)	0.0489 (4)	
F11	0.9283 (3)	0.51416 (7)	0.24445 (8)	0.0562 (4)	
F12	1.12533 (19)	0.48847 (7)	0.34431 (9)	0.0490 (4)	
F13	0.92808 (17)	0.38965 (6)	0.20407 (6)	0.0300 (3)	
F14	1.11525 (16)	0.36215 (6)	0.30559 (7)	0.0284 (3)	
F15	0.53049 (15)	0.16320 (7)	0.06431 (6)	0.0321 (3)	
F16	0.26253 (17)	0.22500 (7)	−0.04917 (6)	0.0322 (3)	
F17	0.17650 (18)	0.11897 (6)	−0.03126 (6)	0.0321 (3)	
F18	0.0027 (2)	0.27674 (7)	0.01901 (7)	0.0436 (3)	
F19	−0.13285 (18)	0.17587 (9)	0.00174 (7)	0.0481 (4)	
F20	−0.00523 (15)	0.25007 (6)	0.15861 (6)	0.0261 (2)	
F21	−0.03121 (15)	0.13923 (6)	0.14101 (7)	0.0276 (3)	
O1	0.54484 (17)	0.15167 (7)	0.43624 (7)	0.0201 (3)	
O2	0.34863 (17)	0.18994 (7)	0.21007 (7)	0.0187 (3)	
O3	0.7469 (2)	0.29766 (7)	0.29659 (8)	0.0249 (3)	
C1	0.4486 (2)	0.12012 (9)	0.48619 (10)	0.0176 (4)	
C2	0.2853 (3)	0.08400 (10)	0.48122 (10)	0.0192 (4)	
C3	0.2388 (3)	0.05780 (10)	0.55538 (11)	0.0223 (4)	
C4	0.3839 (3)	0.09592 (10)	0.61344 (10)	0.0237 (4)	
C5	0.5398 (3)	0.12188 (10)	0.56695 (10)	0.0215 (4)	
C6	0.2748 (2)	0.18792 (9)	0.13720 (10)	0.0172 (4)	
C7	0.3486 (3)	0.17718 (10)	0.07275 (10)	0.0210 (4)	
C8	0.2073 (3)	0.18092 (10)	0.00375 (10)	0.0226 (4)	
C9	0.0244 (3)	0.20780 (12)	0.03398 (11)	0.0276 (4)	
C10	0.0619 (3)	0.19687 (10)	0.12074 (10)	0.0192 (4)	
C11	0.7822 (3)	0.36440 (9)	0.31298 (10)	0.0196 (4)	
C12	0.7004 (3)	0.41170 (10)	0.35294 (11)	0.0234 (4)	
C13	0.7921 (3)	0.48097 (10)	0.35442 (12)	0.0291 (4)	
C14	0.9557 (3)	0.47250 (10)	0.30511 (12)	0.0280 (4)	
C15	0.9490 (3)	0.39496 (10)	0.28019 (10)	0.0210 (4)	
C16	0.4700 (2)	0.14610 (9)	0.35623 (10)	0.0178 (4)	
H16a	0.4625 (2)	0.09673 (9)	0.34028 (10)	0.0213 (4)*	
H16b	0.3401 (2)	0.16670 (9)	0.34662 (10)	0.0213 (4)*	
C17	0.5524 (2)	0.17454 (9)	0.22766 (10)	0.0173 (4)	
H17a	0.5779 (2)	0.12577 (9)	0.21408 (10)	0.0208 (4)*	
H17b	0.6280 (2)	0.20554 (9)	0.19828 (10)	0.0208 (4)*	
C18	0.5989 (3)	0.26292 (9)	0.33273 (10)	0.0190 (4)	
H18a	0.6218 (3)	0.26940 (9)	0.38846 (10)	0.0229 (4)*	
H18b	0.4709 (3)	0.28201 (9)	0.31370 (10)	0.0229 (4)*	
C19	0.6095 (2)	0.18591 (9)	0.31262 (10)	0.0163 (3)	
C20	0.8146 (2)	0.15762 (10)	0.33228 (10)	0.0200 (4)	
H20a	0.8608 (8)	0.1678 (6)	0.3856 (2)	0.0300 (6)*	
H20b	0.8145 (4)	0.10719 (14)	0.3242 (7)	0.0300 (6)*	
H20c	0.8992 (5)	0.1798 (5)	0.2996 (5)	0.0300 (6)*	

### 1,3,3,4,4,5,5-Heptafluoro-2-(3-[(2,3,3,4,4,5,5-heptafluorocyclopenten-1-yl)oxy]-2-{[(2,3,3,4,4,5,5-heptafluorocyclopenten-1-yl)oxy]methyl}-2-methylpropoxy)cyclopentene Atomic displacement parameters (Å^2^)

**Table:**

	*U*^11^	*U*^22^	*U*^33^	*U*^12^	*U*^13^	*U*^23^
F1	0.0214 (5)	0.0330 (6)	0.0232 (6)	−0.0069 (5)	−0.0039 (4)	0.0037 (5)
F2	0.0233 (6)	0.0477 (8)	0.0402 (7)	−0.0013 (5)	0.0156 (5)	−0.0023 (6)
F3	0.0451 (7)	0.0206 (6)	0.0263 (6)	−0.0036 (5)	0.0084 (5)	0.0029 (5)
F4	0.0411 (7)	0.0335 (7)	0.0409 (7)	−0.0024 (6)	0.0230 (6)	−0.0146 (6)
F5	0.0514 (8)	0.0411 (7)	0.0236 (6)	−0.0063 (6)	−0.0031 (5)	0.0101 (5)
F6	0.0387 (7)	0.0368 (7)	0.0247 (6)	−0.0157 (5)	0.0061 (5)	−0.0092 (5)
F7	0.0242 (6)	0.0605 (9)	0.0230 (6)	0.0171 (6)	−0.0028 (5)	−0.0048 (6)
F8	0.0366 (7)	0.0267 (6)	0.0502 (8)	−0.0005 (5)	0.0211 (6)	−0.0091 (6)
F9	0.0435 (8)	0.0204 (6)	0.1018 (13)	0.0097 (6)	0.0048 (8)	0.0101 (7)
F10	0.0689 (10)	0.0416 (8)	0.0381 (8)	−0.0207 (7)	0.0138 (7)	−0.0219 (6)
F11	0.1066 (13)	0.0237 (7)	0.0411 (8)	0.0014 (8)	0.0204 (8)	0.0155 (6)
F12	0.0346 (7)	0.0389 (8)	0.0721 (10)	−0.0132 (6)	0.0020 (7)	−0.0240 (7)
F13	0.0411 (7)	0.0338 (7)	0.0151 (5)	−0.0073 (5)	0.0034 (5)	0.0020 (5)
F14	0.0254 (6)	0.0282 (6)	0.0307 (6)	0.0003 (5)	0.0002 (5)	0.0029 (5)
F15	0.0181 (5)	0.0552 (8)	0.0241 (6)	0.0040 (5)	0.0071 (4)	−0.0070 (5)
F16	0.0359 (6)	0.0416 (7)	0.0197 (6)	−0.0083 (5)	0.0063 (5)	0.0070 (5)
F17	0.0421 (7)	0.0334 (7)	0.0202 (6)	−0.0071 (5)	0.0017 (5)	−0.0064 (5)
F18	0.0531 (8)	0.0464 (8)	0.0319 (7)	0.0215 (7)	0.0074 (6)	0.0164 (6)
F19	0.0238 (6)	0.0941 (12)	0.0253 (6)	−0.0156 (7)	−0.0009 (5)	−0.0091 (7)
F20	0.0235 (5)	0.0290 (6)	0.0264 (6)	0.0072 (5)	0.0047 (4)	−0.0029 (5)
F21	0.0226 (5)	0.0294 (6)	0.0316 (6)	−0.0074 (5)	0.0064 (5)	0.0031 (5)
O1	0.0202 (6)	0.0251 (7)	0.0148 (6)	−0.0052 (5)	0.0014 (5)	0.0013 (5)
O2	0.0167 (6)	0.0251 (7)	0.0139 (6)	0.0018 (5)	0.0008 (5)	−0.0010 (5)
O3	0.0336 (7)	0.0160 (6)	0.0279 (7)	−0.0067 (6)	0.0146 (6)	−0.0047 (5)
C1	0.0179 (8)	0.0187 (8)	0.0167 (9)	0.0029 (7)	0.0036 (7)	0.0011 (7)
C2	0.0171 (8)	0.0213 (9)	0.0183 (9)	0.0011 (7)	−0.0006 (7)	0.0001 (7)
C3	0.0211 (9)	0.0226 (9)	0.0244 (10)	0.0010 (7)	0.0079 (7)	0.0003 (8)
C4	0.0306 (10)	0.0238 (10)	0.0175 (9)	0.0019 (8)	0.0064 (8)	−0.0005 (7)
C5	0.0187 (9)	0.0267 (10)	0.0187 (9)	0.0010 (7)	0.0012 (7)	−0.0036 (8)
C6	0.0184 (8)	0.0170 (8)	0.0159 (8)	−0.0009 (7)	0.0012 (7)	0.0008 (7)
C7	0.0176 (8)	0.0265 (10)	0.0194 (9)	−0.0002 (7)	0.0042 (7)	−0.0009 (7)
C8	0.0259 (9)	0.0283 (10)	0.0142 (8)	−0.0044 (8)	0.0050 (7)	0.0005 (7)
C9	0.0211 (9)	0.0411 (12)	0.0197 (10)	0.0005 (8)	−0.0009 (7)	0.0026 (8)
C10	0.0186 (9)	0.0216 (9)	0.0179 (9)	−0.0001 (7)	0.0045 (7)	−0.0004 (7)
C11	0.0266 (9)	0.0153 (9)	0.0162 (8)	−0.0025 (7)	0.0001 (7)	0.0004 (7)
C12	0.0263 (9)	0.0199 (9)	0.0244 (10)	−0.0006 (8)	0.0042 (8)	−0.0005 (8)
C13	0.0358 (11)	0.0162 (9)	0.0342 (11)	0.0024 (8)	0.0009 (9)	−0.0031 (8)
C14	0.0366 (11)	0.0192 (10)	0.0274 (10)	−0.0065 (8)	0.0015 (8)	0.0037 (8)
C15	0.0271 (9)	0.0193 (9)	0.0162 (9)	−0.0022 (7)	0.0012 (7)	0.0022 (7)
C16	0.0191 (8)	0.0196 (9)	0.0139 (8)	−0.0026 (7)	−0.0009 (7)	0.0004 (7)
C17	0.0146 (8)	0.0201 (9)	0.0171 (8)	0.0011 (7)	0.0013 (6)	−0.0011 (7)
C18	0.0212 (9)	0.0170 (9)	0.0198 (9)	−0.0035 (7)	0.0061 (7)	−0.0012 (7)
C19	0.0176 (8)	0.0161 (9)	0.0151 (8)	−0.0007 (7)	0.0015 (6)	−0.0002 (7)
C20	0.0184 (8)	0.0215 (9)	0.0195 (9)	0.0009 (7)	0.0004 (7)	−0.0003 (7)

### 1,3,3,4,4,5,5-Heptafluoro-2-(3-[(2,3,3,4,4,5,5-heptafluorocyclopenten-1-yl)oxy]-2-{[(2,3,3,4,4,5,5-heptafluorocyclopenten-1-yl)oxy]methyl}-2-methylpropoxy)cyclopentene Geometric parameters (Å, º)

**Table:**

F1—C2	1.335 (2)	C1—C5	1.495 (2)
F2—C3	1.341 (2)	C2—C3	1.485 (3)
F3—C3	1.364 (2)	C3—C4	1.540 (3)
F4—C4	1.347 (2)	C4—C5	1.536 (3)
F5—C4	1.343 (2)	C6—C7	1.331 (3)
F6—C5	1.349 (2)	C6—C10	1.497 (2)
F7—C5	1.354 (2)	C7—C8	1.477 (3)
F8—C12	1.334 (2)	C8—C9	1.543 (3)
F9—C13	1.356 (2)	C9—C10	1.544 (3)
F10—C13	1.345 (3)	C11—C12	1.328 (3)
F11—C14	1.336 (2)	C11—C15	1.494 (3)
F12—C14	1.337 (2)	C12—C13	1.478 (3)
F13—C15	1.345 (2)	C13—C14	1.540 (3)
F14—C15	1.354 (2)	C14—C15	1.554 (3)
F15—C7	1.333 (2)	C16—H16a	0.9900
F16—C8	1.358 (2)	C16—H16b	0.9900
F17—C8	1.348 (2)	C16—C19	1.530 (2)
F18—C9	1.357 (3)	C17—H17a	0.9900
F19—C9	1.328 (2)	C17—H17b	0.9900
F20—C10	1.342 (2)	C17—C19	1.527 (2)
F21—C10	1.359 (2)	C18—H18a	0.9900
O1—C1	1.329 (2)	C18—H18b	0.9900
O1—C16	1.454 (2)	C18—C19	1.527 (2)
O2—C6	1.332 (2)	C19—C20	1.538 (2)
O2—C17	1.457 (2)	C20—H20a	0.9800
O3—C11	1.332 (2)	C20—H20b	0.9800
O3—C18	1.453 (2)	C20—H20c	0.9800
C1—C2	1.334 (3)		
			
C16—O1—C1	117.84 (13)	C15—C11—C12	110.70 (16)
C17—O2—C6	116.93 (13)	C11—C12—F8	127.44 (17)
C18—O3—C11	118.06 (14)	C13—C12—F8	118.43 (17)
C2—C1—O1	134.45 (17)	C13—C12—C11	114.13 (17)
C5—C1—O1	115.95 (15)	F10—C13—F9	105.80 (17)
C5—C1—C2	109.57 (16)	C12—C13—F9	111.57 (17)
C1—C2—F1	127.48 (17)	C12—C13—F10	113.02 (18)
C3—C2—F1	118.77 (15)	C14—C13—F9	110.79 (18)
C3—C2—C1	113.75 (16)	C14—C13—F10	111.31 (17)
F3—C3—F2	105.90 (15)	C14—C13—C12	104.47 (16)
C2—C3—F2	112.73 (16)	F12—C14—F11	108.02 (17)
C2—C3—F3	112.67 (15)	C13—C14—F11	110.69 (18)
C4—C3—F2	112.63 (15)	C13—C14—F12	111.12 (17)
C4—C3—F3	109.73 (15)	C15—C14—F11	110.45 (16)
C4—C3—C2	103.32 (15)	C15—C14—F12	111.18 (17)
F5—C4—F4	107.25 (15)	C15—C14—C13	105.40 (15)
C3—C4—F4	110.08 (16)	F14—C15—F13	106.17 (15)
C3—C4—F5	112.23 (16)	C11—C15—F13	111.80 (15)
C5—C4—F4	109.66 (16)	C11—C15—F14	111.93 (15)
C5—C4—F5	113.06 (16)	C14—C15—F13	110.77 (15)
C5—C4—C3	104.57 (14)	C14—C15—F14	111.00 (15)
F7—C5—F6	107.20 (15)	C14—C15—C11	105.26 (15)
C1—C5—F6	112.84 (16)	H16a—C16—O1	110.45 (9)
C1—C5—F7	110.89 (15)	H16b—C16—O1	110.45 (9)
C4—C5—F6	111.66 (15)	H16b—C16—H16a	108.6
C4—C5—F7	109.16 (16)	C19—C16—O1	106.40 (13)
C4—C5—C1	105.08 (14)	C19—C16—H16a	110.45 (9)
C7—C6—O2	133.91 (17)	C19—C16—H16b	110.45 (9)
C10—C6—O2	116.23 (15)	H17a—C17—O2	110.09 (9)
C10—C6—C7	109.82 (16)	H17b—C17—O2	110.09 (9)
C6—C7—F15	127.73 (17)	H17b—C17—H17a	108.4
C8—C7—F15	117.98 (15)	C19—C17—O2	108.07 (13)
C8—C7—C6	114.29 (16)	C19—C17—H17a	110.09 (9)
F17—C8—F16	105.98 (14)	C19—C17—H17b	110.09 (9)
C7—C8—F16	112.53 (16)	H18a—C18—O3	110.44 (9)
C7—C8—F17	113.01 (16)	H18b—C18—O3	110.44 (9)
C9—C8—F16	110.84 (16)	H18b—C18—H18a	108.6
C9—C8—F17	111.36 (16)	C19—C18—O3	106.45 (14)
C9—C8—C7	103.24 (15)	C19—C18—H18a	110.44 (9)
F19—C9—F18	107.48 (17)	C19—C18—H18b	110.44 (9)
C8—C9—F18	109.68 (16)	C17—C19—C16	108.89 (14)
C8—C9—F19	112.29 (17)	C18—C19—C16	108.24 (14)
C10—C9—F18	109.21 (16)	C18—C19—C17	110.82 (14)
C10—C9—F19	113.19 (16)	C20—C19—C16	110.53 (14)
C10—C9—C8	104.95 (15)	C20—C19—C17	107.11 (14)
F21—C10—F20	106.06 (14)	C20—C19—C18	111.24 (14)
C6—C10—F20	113.34 (15)	H20a—C20—C19	109.5
C6—C10—F21	110.95 (15)	H20b—C20—C19	109.5
C9—C10—F20	111.73 (15)	H20b—C20—H20a	109.5
C9—C10—F21	110.42 (15)	H20c—C20—C19	109.5
C9—C10—C6	104.44 (14)	H20c—C20—H20a	109.5
C12—C11—O3	134.00 (18)	H20c—C20—H20b	109.5
C15—C11—O3	115.30 (16)		
			
F1—C2—C1—O1	0.3 (3)	F14—C15—C14—C13	−119.26 (16)
F1—C2—C1—C5	−177.52 (19)	F15—C7—C6—O2	1.5 (3)
F1—C2—C3—F2	−48.42 (18)	F15—C7—C6—C10	−176.0 (2)
F1—C2—C3—F3	71.38 (17)	F15—C7—C8—F16	−52.69 (18)
F1—C2—C3—C4	−170.27 (17)	F15—C7—C8—F17	67.32 (18)
F2—C3—C2—C1	132.64 (16)	F15—C7—C8—C9	−172.25 (19)
F2—C3—C4—F4	−21.91 (17)	F16—C8—C7—C6	127.91 (17)
F2—C3—C4—F5	97.46 (16)	F16—C8—C9—F18	−19.40 (16)
F2—C3—C4—C5	−139.63 (16)	F16—C8—C9—F19	100.03 (16)
F3—C3—C2—C1	−107.57 (17)	F16—C8—C9—C10	−136.61 (16)
F3—C3—C4—F4	−139.60 (14)	F17—C8—C7—C6	−112.08 (16)
F3—C3—C4—F5	−20.24 (16)	F17—C8—C9—F18	−137.13 (15)
F3—C3—C4—C5	102.68 (15)	F17—C8—C9—F19	−17.70 (17)
F4—C4—C3—C2	100.02 (16)	F17—C8—C9—C10	105.67 (16)
F4—C4—C5—F6	23.43 (17)	F18—C9—C8—C7	101.32 (16)
F4—C4—C5—F7	141.78 (15)	F18—C9—C10—F20	23.24 (17)
F4—C4—C5—C1	−99.23 (16)	F18—C9—C10—F21	141.03 (15)
F5—C4—C3—C2	−140.61 (16)	F18—C9—C10—C6	−99.64 (16)
F5—C4—C5—F6	−96.19 (16)	F19—C9—C8—C7	−139.25 (18)
F5—C4—C5—F7	22.16 (17)	F19—C9—C10—F20	−96.44 (18)
F5—C4—C5—C1	141.15 (16)	F19—C9—C10—F21	21.34 (19)
F6—C5—C1—O1	46.89 (17)	F19—C9—C10—C6	140.67 (18)
F6—C5—C1—C2	−134.85 (16)	F20—C10—C6—O2	46.61 (17)
F6—C5—C4—C3	141.43 (16)	F20—C10—C6—C7	−135.38 (16)
F7—C5—C1—O1	−73.39 (17)	F20—C10—C9—C8	140.77 (16)
F7—C5—C1—C2	104.87 (17)	F21—C10—C6—O2	−72.58 (15)
F7—C5—C4—C3	−100.21 (15)	F21—C10—C6—C7	105.42 (16)
F8—C12—C11—O3	0.8 (3)	F21—C10—C9—C8	−101.44 (16)
F8—C12—C11—C15	−179.4 (2)	O1—C1—C2—C3	179.1 (2)
F8—C12—C13—F9	−59.5 (2)	O1—C1—C5—C4	168.79 (17)
F8—C12—C13—F10	59.58 (19)	O1—C16—C19—C17	173.11 (13)
F8—C12—C13—C14	−179.26 (19)	O1—C16—C19—C18	−66.34 (15)
F9—C13—C12—C11	120.75 (19)	O1—C16—C19—C20	55.72 (15)
F9—C13—C14—F11	−2.68 (19)	O2—C6—C7—C8	−179.2 (2)
F9—C13—C14—F12	117.35 (17)	O2—C6—C10—C9	168.45 (17)
F9—C13—C14—C15	−122.11 (17)	O2—C17—C19—C16	50.98 (15)
F10—C13—C12—C11	−120.16 (18)	O2—C17—C19—C18	−67.96 (15)
F10—C13—C14—F11	−120.11 (17)	O2—C17—C19—C20	170.53 (14)
F10—C13—C14—F12	−0.08 (18)	O3—C11—C12—C13	−179.5 (2)
F10—C13—C14—C15	120.46 (17)	O3—C11—C15—C14	178.33 (17)
F11—C14—C13—C12	117.60 (18)	O3—C18—C19—C16	174.23 (13)
F11—C14—C15—F13	3.45 (19)	O3—C18—C19—C17	−66.43 (15)
F11—C14—C15—F14	121.15 (17)	O3—C18—C19—C20	52.62 (15)
F11—C14—C15—C11	−117.56 (18)	C1—C2—C3—C4	10.78 (17)
F12—C14—C13—C12	−122.37 (17)	C1—C5—C4—C3	18.77 (16)
F12—C14—C15—F13	−116.45 (17)	C2—C3—C4—C5	−17.70 (15)
F12—C14—C15—F14	1.24 (18)	C6—C7—C8—C9	8.35 (19)
F12—C14—C15—C11	122.54 (17)	C6—C10—C9—C8	17.88 (15)
F13—C15—C11—O3	58.00 (17)	C7—C8—C9—C10	−15.89 (16)
F13—C15—C11—C12	−121.86 (17)	C11—C12—C13—C14	1.00 (19)
F13—C15—C14—C13	123.05 (16)	C11—C15—C14—C13	2.03 (16)
F14—C15—C11—O3	−60.98 (16)	C12—C13—C14—C15	−1.83 (17)
F14—C15—C11—C12	119.16 (16)		
